# Draft Metagenome Sequences of the *Sphagnum* (Peat Moss) Microbiome from Ambient and Warmed Environments across Europe

**DOI:** 10.1128/mra.00400-22

**Published:** 2022-09-07

**Authors:** Bryan T. Piatkowski, Dana L. Carper, Alyssa A. Carrell, I-Min A. Chen, Alicia Clum, Chris Daum, Emiley A. Eloe-Fadrosh, Daniel Gilbert, Gustaf Granath, Marcel Huntemann, Sara S. Jawdy, Ingeborg Jenneken Klarenberg, Joel E. Kostka, Nikos C. Kyrpides, Travis J. Lawrence, Supratim Mukherjee, Mats B. Nilsson, Krishnaveni Palaniappan, Dale A. Pelletier, Christa Pennacchio, T. B. K. Reddy, Simon Roux, A. Jonathan Shaw, Denis Warshan, Tatjana Živković, David J. Weston

**Affiliations:** a Biosciences Division, Oak Ridge National Laboratory, Oak Ridge, Tennessee, USA; b Department of Energy Joint Genome Institute, Berkeley, California, USA; c Laboratoire Chrono-Environnement/UMR 6249, CNRS, University of Bourgogne Franche-Comté, Montbéliard, France; d Department of Ecology and Genetics, Uppsala University, Uppsala, Sweden; e University of Akureyri, Akureyri, Iceland; f Georgia Institute of Technology, Atlanta, Georgia, USA; g Department of Forest Ecology and Management, Swedish University of Agricultural Sciences, Umeå, Sweden; h Department of Biology, Duke University, Durham, North Carolina, USA; i Faculty of Life and Environmental Sciences, University of Iceland, Reykjavik, Iceland; j Department of Biology, Dalhousie University, Halifax, Nova Scotia, Canada; University of California, Riverside

## Abstract

We present 49 metagenome assemblies of the microbiome associated with *Sphagnum* (peat moss) collected from ambient, artificially warmed, and geothermally warmed conditions across Europe. These data will enable further research regarding the impact of climate change on plant-microbe symbiosis, ecology, and ecosystem functioning of northern peatland ecosystems.

## ANNOUNCEMENT

Peat mosses (*Sphagnum* spp.) are keystone species of northern peatlands and have an extraordinary impact on global biogeochemical cycles. These northern latitude ecosystems harbor over one-quarter of terrestrial carbon in the form of peat, or incompletely decomposed biomass, but cover roughly 3% of Earth’s land mass (1). Furthermore, peatlands store approximately 9 to 16% of global soil nitrogen (2, 3), which is largely derived from the biological fixation of atmospheric nitrogen by microbes that live in symbiosis with peat mosses (4, 5). Recently, much effort has been dedicated to understanding how *Sphagnum* will respond to projected scenarios of climate change (e.g., see references 6 and 7), but gaps exist in our knowledge about how such changes might affect the community composition and functioning of the *Sphagnum* microbiome. Here, we present microbiome metagenome assemblies for 49 samples of *Sphagnum*, representing 11 species collected from ambient, artificially warmed, and geothermally warmed environmental conditions.

**FIG 1:**
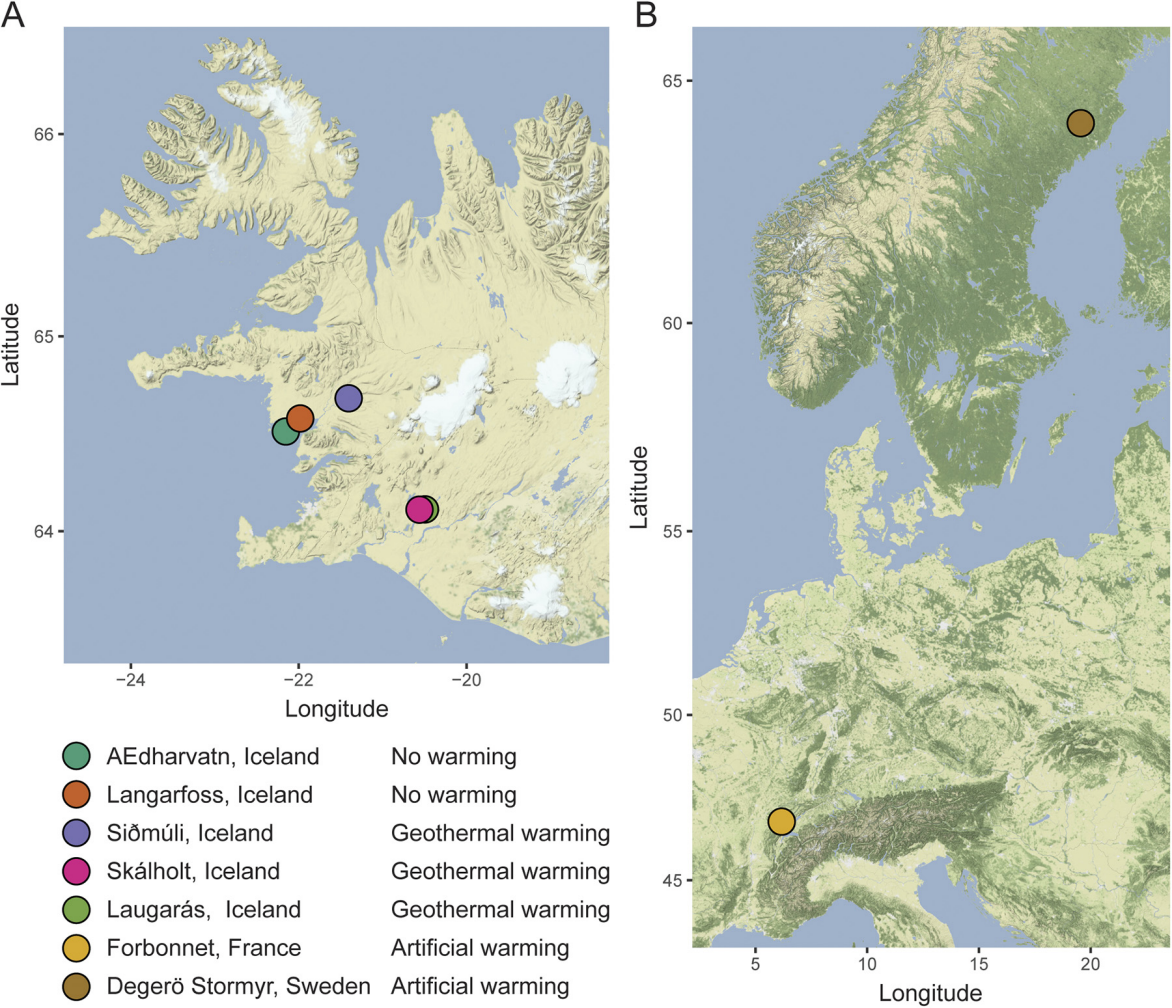
Collection sites for *Sphagnum* metagenome samples. (A) Plants at Icelandic sites were collected from only ambient or both ambient and geothermally warmed conditions. (B) Plants in France and Sweden were collected from both ambient and artificially warmed conditions.

Samples were collected from seven sites across three countries in Europe (Fig. 1), and the dominant species of *Sphagnum* were sampled at each site. Artificial warming experiments have been conducted since 1994 at the Degerö Stormyr peatland in Sweden (8) and since 2008 at the Forbonnet peatland in France (9). The average temperatures of moss samples were 12.2°C under ambient conditions, 14.7°C under artificially warmed conditions, and 27.2°C under geothermally warmed conditions (Table 1). Samples in the Sphagnum magellanicum species complex, i.e., Sphagnum divinum and Sphagnum medium, were identified on the basis of morphological and genetic analyses.

**TABLE 1:** Properties of the *Sphagnum* metagenomes

*Sphagnum* species	Sample name	Country of isolation	Region of isolation	Temp treatment	Moss temp (°C)	IMG genome identification no.	ENA sample accession no.	ENA run accession no.	ENA analysis accession no.	No. of filtered reads	Genome size (bp)	No. of contigs	Contig *N*_50_ (bp)	Contig *L*_50_ (bp)	Avg coverage of assembled sequences (×)	No. of genes	No. of genome bins	Estimated no. of genomes
S. angustifolium	NH_ANG_ICE	Iceland	Langarfoss	None	10.0	3300047495	ERS12393009	ERR9963747	ERZ12298656	1.3E+08	1.2E+09	1.4E+06	1.7E+05	1,287	9.2	2.2E+06	12	172
S. auriculatum	1_AUR_ICE	Iceland	Laugarás	Geothermal	34.0	3300047328	ERS12393010	ERR9963748	ERZ12293583	1.1E+08	1.4E+09	1.2E+06	1.4E+05	2,092	9.1	2.1E+06	31	140
S. auriculatum	3_AUR_ICE	Iceland	Laugarás	Geothermal	22.0	3300047494	ERS12393011	ERR9963749	ERZ12293588	1.3E+08	1.5E+09	1.6E+06	1.7E+05	1,266	9.8	2.3E+06	56	235
S. auriculatum	5_AUR_ICE	Iceland	Laugarás	Geothermal	27.0	3300047169	ERS12393012	ERR9963750	ERZ12293627	1.1E+08	1.3E+09	1.4E+06	2.5E+05	1,291	9.6	2.0E+06	22	105
S. balticum	16OTC_BA_DE	Sweden	Degerö Stormyr	Artificial	18.1	3300047167	ERS12393013	ERR9963751	ERZ12293049	1.1E+08	1.2E+09	1.6E+06	2.0E+05	1,112	8.5	2.3E+06	6	150
S. balticum	11C_BA_DE	Sweden	Degerö Stormyr	None	14.8	3300047166	ERS12393014	ERR9963752	ERZ12293046	1.0E+08	1.3E+09	1.7E+06	2.7E+05	1,053	8.0	2.4E+06	19	195
S. balticum	19C_BA_DE	Sweden	Degerö Stormyr	None	14.8	3300047165	ERS12393015	ERR9963753	ERZ12298654	1.2E+08	1.4E+09	1.8E+06	3.1E+05	968	8.2	2.6E+06	16	207
S. divinum	2OTC_MAG_FR	France	Forbonnet	Artificial	13.4	3300047335	ERS12393016	ERR9963754	ERZ12293584	1.4E+08	1.5E+09	2.1E+06	3.9E+05	853	7.3	2.9E+06	14	238
S. divinum	3OTC_MAG_FR	France	Forbonnet	Artificial	13.4	3300047332	ERS12393017	ERR9963755	ERZ12295337	1.3E+08	1.2E+09	1.9E+06	4.2E+05	633	7.0	2.4E+06	12	279
S. divinum	6OTC_MAG_FR	France	Forbonnet	Artificial	13.4	3300047324	ERS12393018	ERR9963756	ERZ12293629	1.4E+08	1.5E+09	2.3E+06	4.6E+05	728	7.8	2.9E+06	17	257
S. divinum	1C_MAG_FR	France	Forbonnet	None	11.4	3300047326	ERS12393019	ERR9963757	ERZ12293615	1.3E+08	1.5E+09	2.0E+06	3.3E+05	919	8.2	2.7E+06	17	237
S. divinum	4C_MAG_FR	France	Forbonnet	None	11.4	3300047325	ERS12393020	ERR9963758	ERZ12293589	1.6E+08	1.6E+09	2.3E+06	4.5E+05	767	7.9	3.1E+06	15	324
S. divinum	5C_MAG_FR	France	Forbonnet	None	11.4	3300047175	ERS12393021	ERR9963759	ERZ12293624	1.3E+08	1.4E+09	1.9E+06	2.9E+05	959	9.0	2.5E+06	21	234
S. fallax	10OTC_SF_FR	France	Forbonnet	Artificial	13.4	3300047177	ERS12393022	ERR9963760	ERZ12293045	9.4E+07	1.0E+09	1.5E+06	3.2E+05	776	7.6	1.9E+06	7	170
S. fallax	12OTC_SF_FR	France	Forbonnet	Artificial	13.4	3300047174	ERS12393023	ERR9963761	ERZ12293614	1.3E+08	1.3E+09	1.6E+06	2.7E+05	1,054	9.5	2.3E+06	16	208
S. fallax	3OTC_SF_FR	France	Forbonnet	Artificial	13.4	3300047176	ERS12393024	ERR9963762	ERZ12293587	9.6E+07	9.0E+08	1.0E+06	1.5E+05	1,310	8.3	1.5E+06	9	104
S. fallax	6OTC_SF_FR	France	Forbonnet	Artificial	13.4	3300047333	ERS12393025	ERR9963763	ERZ12297591	1.3E+08	1.4E+09	2.1E+06	4.1E+05	809	7.7	2.8E+06	9	233
S. fallax	8OTC_SF_FR	France	Forbonnet	Artificial	13.4	3300047179	ERS12393026	ERR9963764	ERZ12298564	1.3E+08	1.3E+09	1.7E+06	2.3E+05	1,075	10.8	2.4E+06	5	169
S. fallax	11C_SF_FR	France	Forbonnet	None	11.4	3300047178	ERS12393027	ERR9963765	ERZ12293047	1.3E+08	1.3E+09	1.9E+06	3.4E+05	844	9.1	2.5E+06	21	255
S. fallax	1C_SF_FR	France	Forbonnet	None	11.4	3300049214	ERS12393028	ERR9963766	ERZ12293582	1.2E+08	1.4E+09	1.8E+06	2.8E+05	1,004	8.7	2.6E+06	13	204
S. fallax	4C_SF_FR	France	Forbonnet	None	11.4	3300047330	ERS12393029	ERR9963767	ERZ12293621	1.4E+08	1.4E+09	1.5E+06	1.4E+05	1,350	10.7	2.3E+06	7	171
S. fallax	5C_SF_FR	France	Forbonnet	None	11.4	3300047329	ERS12393030	ERR9963768	ERZ12295550	1.3E+08	1.3E+09	2.0E+06	4.0E+05	819	8.1	2.4E+06	7	174
S. fallax	7C_SF_FR	France	Forbonnet	None	11.4	3300047334	ERS12393031	ERR9963769	ERZ12298487	1.9E+08	2.0E+09	2.5E+06	3.9E+05	1,032	9.4	3.6E+06	18	341
S. fallax	9C_SF_FR	France	Forbonnet	None	11.4	3300047331	ERS12393032	ERR9963770	ERZ12293631	1.1E+08	1.1E+09	1.3E+06	1.8E+05	1,146	9.0	1.9E+06	4	124
S. girgensohnii	12_GIR_ICE	Iceland	Skálholt	Geothermal	20.0	3300047171	ERS12393033	ERR9963771	ERZ12295332	1.1E+08	9.9E+08	6.4E+05	3.1E+04	5,777	14.1	1.4E+06	28	111
S. lindbergii	16OTC_LI_DE	Sweden	Degerö Stormyr	Artificial	18.1	3300047164	ERS12393034	ERR9963772	ERZ12298653	1.2E+08	1.3E+09	1.3E+06	1.2E+05	1,894	9.2	2.2E+06	15	183
S. lindbergii	4OTC_LI_DE	Sweden	Degerö Stormyr	Artificial	18.1	3300047173	ERS12393035	ERR9963773	ERZ12298655	1.4E+08	1.4E+09	1.6E+06	2.2E+05	1,153	9.0	2.5E+06	26	265
S. lindbergii	11C_LI_DE	Sweden	Degerö Stormyr	None	14.8	3300047473	ERS12393036	ERR9963774	ERZ12295331	9.4E+07	1.2E+09	2.0E+06	4.5E+05	615	7.5	2.5E+06	12	212
S. lindbergii	19C_LI_DE	Sweden	Degerö Stormyr	None	14.8	3300047327	ERS12393037	ERR9963775	ERZ12295333	1.1E+08	1.1E+09	8.1E+05	3.7E+04	4,496	10.3	1.7E+06	19	146
S. medium	H_MAG_ICE	Iceland	Siðmúli	Geothermal	28.0	3300047499	ERS12393038	ERR9963776	ERZ12295052	1.5E+08	1.6E+09	1.3E+06	8.6E+04	2,974	11.4	2.4E+06	67	206
S. medium	H1_MAG_ICE	Iceland	Siðmúli	Geothermal	28.0	3300049255	ERS12393039	ERR9963777	ERZ12298643	5.4E+08	2.6E+09	1.7E+06	4.4E+04	7,822	29.3	3.1E+06	158	335
S. medium	H2_MAG_ICE	Iceland	Siðmúli	Geothermal	28.0	3300048014	ERS12393040	ERR9963778	ERZ12294360	3.6E+08	2.4E+09	2.3E+06	1.4E+05	2,584	19.8	3.0E+06	96	290
S. medium	H3_MAG_ICE	Iceland	Siðmúli	Geothermal	28.0	3300048015	ERS12393041	ERR9963779	ERZ12298651	3.4E+08	2.3E+09	2.7E+06	1.8E+05	1,940	19.6	3.0E+06	77	301
S. medium	C_MAG_ICE	Iceland	Siðmúli	None	12.0	3300047493	ERS12393042	ERR9963780	ERZ12293733	1.2E+08	1.5E+09	1.5E+06	1.9E+05	1,636	8.3	2.5E+06	21	222
S. medium	C1_MAG_ICE	Iceland	Siðmúli	None	12.0	3300049217	ERS12393043	ERR9963781	ERZ12293632	4.6E+08	2.3E+09	3.4E+06	2.9E+05	1,361	24.7	3.2E+06	61	319
S. medium	C2_MAG_ICE	Iceland	Siðmúli	None	12.0	3300049218	ERS12393044	ERR9963782	ERZ12298641	3.7E+08	2.3E+09	2.4E+06	1.4E+05	2,432	20.9	2.9E+06	86	283
S. medium	C3_MAG_ICE	Iceland	Siðmúli	None	12.0	3300049617	ERS12393045	ERR9963783	ERZ12293637	3.7E+08	2.3E+09	2.6E+06	1.8E+05	1,957	19.9	3.0E+06	69	304
S. subnitens	SUB_ICE	Iceland	Langarfoss	None	10.0	3300047170	ERS12393046	ERR9963784	ERZ12295330	1.2E+08	1.3E+09	1.8E+06	3.7E+05	796	7.8	2.4E+06	16	227
S. teres	NH_TE_ICE	Iceland	AEdharvatn	None	10.0	3300047498	ERS12393047	ERR9963785	ERZ12295321	1.4E+08	1.4E+09	1.7E+06	2.5E+05	1,195	7.6	2.5E+06	6	166
S. teres	H_TE_ICE	Iceland	Siðmúli	Geothermal	28.0	3300047172	ERS12393048	ERR9963786	ERZ12298652	1.1E+08	1.2E+09	1.2E+06	1.2E+05	1,512	9.9	1.9E+06	54	206
S. teres	H1_TE_ICE	Iceland	Siðmúli	Geothermal	28.0	3300047462	ERS12393049	ERR9963787	ERZ12298644	5.1E+08	2.9E+09	2.8E+06	1.5E+05	2,554	23.6	3.7E+06	152	406
S. teres	H2_TE_ICE	Iceland	Siðmúli	Geothermal	28.0	3300049215	ERS12393050	ERR9963788	ERZ12294693	4.2E+08	2.1E+09	2.2E+06	1.0E+05	2,547	27.0	2.7E+06	154	387
S. teres	C_TE_ICE	Iceland	Siðmúli	None	12.0	3300047168	ERS12393051	ERR9963789	ERZ12293768	1.3E+08	1.3E+09	1.1E+06	3.6E+04	3,842	10.8	2.0E+06	38	190
S. teres	C1_TE_ICE	Iceland	Siðmúli	None	12.0	3300047994	ERS12393052	ERR9963790	ERZ12293633	3.6E+08	2.4E+09	2.8E+06	1.7E+05	2,009	18.8	3.1E+06	119	392
S. teres	C2_TE_ICE	Iceland	Siðmúli	None	12.0	3300047678	ERS12393053	ERR9963791	ERZ12298642	3.5E+08	2.5E+09	3.3E+06	3.6E+05	1,424	16.4	3.4E+06	127	475
S. teres	C3_TE_ICE	Iceland	Siðmúli	None	12.0	3300049216	ERS12393054	ERR9963792	ERZ12293692	4.5E+08	2.6E+09	2.5E+06	1.2E+05	2,899	23.2	3.2E+06	129	394
S. teres	7_TE_ICE	Iceland	Skálholt	Geothermal	34.0	3300047497	ERS12393055	ERR9963793	ERZ12293630	1.3E+08	1.3E+09	8.2E+05	4.4E+04	5,424	10.9	1.9E+06	76	217
S. teres	9_TE_ICE	Iceland	Skálholt	None	16.0	3300047496	ERS12393056	ERR9963794	ERZ12298640	1.3E+08	1.4E+09	1.6E+06	2.2E+05	1,301	7.6	2.5E+06	28	274
S. warnstorfii	11_WAR_ICE	Iceland	Skálholt	Geothermal	20.0	3300047163	ERS12393057	ERR9963795	ERZ12293048	1.2E+08	1.3E+09	1.8E+06	3.2E+05	868	7.7	2.5E+06	30	235

Samples were flash frozen in the field using liquid nitrogen. Genomic DNA extractions were performed using DNeasy Plant Pro DNA extraction kits (Qiagen) according to the manufacturer’s instructions. Illumina library construction using KAPA DNA library kits and 2 × 151-bp sequencing using the Illumina NovaSeq S4 platform were performed by the U.S. Department of Energy’s (DOE) Joint Genome Institute (JGI). All metagenomes were processed by the DOE JGI Metagenome Workflow (10). In brief, reads were quality controlled using the BBTools package v38.92 (https://bbtools.jgi.doe.gov), metagenomes were assembled using metaSPAdes v3.15.2 (11), and the assemblies were annotated using the IMG Annotation Pipeline v5.0.24 (10). MetaBAT v0.32.4 (12) was used to identify genome bins, with a 3-kb minimum contig cutoff value, contig coverage information included, and the –superspecific parameter enforced. Genome bins of at least medium quality were kept according to the minimum information on a metagenome-assembled genome (MIMAG) standards (13). Relevant statistics for these metagenome assemblies and annotations can be found in Table 1. Metadata and annotations can be obtained through the Genome OnLine Database (GOLD study identification number Gs0154043) (14) and Integrated Microbial Genomes and Microbiomes (IMG/M) online system (15), respectively.

### Data availability.

The data have been deposited in the European Nucleotide Archive (ENA) at EMBL-EBI (project number PRJEB54621).
